# Role of LPSO Phase in Crack Propagation Behavior of an As-Cast Mg-Y-Zn Alloy Subjected to Dynamic Loadings

**DOI:** 10.3390/ma12030498

**Published:** 2019-02-06

**Authors:** Xuezhi Shi, Yunqian Long, Huiqiu Zhang, Liqiao Chen, Yingtang Zhou, Xiaoming Yu, Xuan Yu, Lu Cai, Zhe Leng

**Affiliations:** Innovation and Application Institute, Zhejiang Ocean University, Zhoushan 316022, China; shixuezhi@zjou.edu.cn (X.S.); longyunqian@zjou.edu.cn (Y.L.); zhanghuiqiu@zjou.edu.cn (H.Z.); chenliqiao@zjou.edu.cn (L.C.); zhouyingtang@zjou.edu.cn (Y.Z.); yuxiaoming@zjou.edu.cn (X.Y.); yuxuan@zjou.edu.cn (X.Y.); lucai89@126.com (L.C.)

**Keywords:** Mg alloy, LPSO phase, crack propagation, dynamic loading

## Abstract

In this work, the role of long period stacking ordered (LPSO) phase in the crack propagation behavior of an as-cast Mg_95.5_Y_3_Zn_1.5_ alloy was investigated by dynamic four-point bent tests. The as-cast Mg_95.5_Y_3_Zn_1.5_ alloy is mainly composed of Mg matrix, 18R LPSO phase located at the grain boundaries and 14H LPSO phase located within the Mg matrix. The alloy exhibits excellent dynamic mechanical properties; the yield stress, maximum stress and strain to failure are 190.51 ± 3.52 MPa, 378.32 ± 4.26 MPa and 0.168 ± 0.006, respectively, at the strain rate of ~3000 s^−1^. The LPSO phase effectively hinders dynamic crack propagation in four typical ways, including crack tip blunting, crack opening inhibition, crack deflection and crack bridging, which are beneficial to the mechanical properties of the alloy under dynamic loadings.

## 1. Introduction

Being the lightest metal structural materials, Mg alloys have shown splendid prospects in the fields of electricity, automobile and aerospace industries. Unfortunately, their relative low strength and poor ductility greatly impeded their further utilization [1,2]. To break through the bottleneck, a series of new Mg-based alloys have been designed and some positive results have been obtained [3,4,5,6,7,8]. Among them, Mg-RE-Zn alloys containing long period stacking ordered (LPSO) phase have received a considerable attention since the discovery of Mg_97_Y_2_Zn_1_ (at.%) alloy, which is produced by rapidly solidified powder metallurgy (RS P/M) technology. The alloy exhibits an excellent yield stress of ~610 MPa, which is about 2.5 times higher than that of conventional Mg alloys. Meanwhile, the elongation of the alloy reaches up to ~5% at room temperature [9]. One of the key factors for such superior mechanical properties is the formation of LPSO phase [9,10,11,12]. So far, a series of LPSO-Mg alloys with superior properties have been developed [13,14,15]. For example, Kawamura et al. [13] prepared an extruded LPSO Mg_97_Y_2_Cu_1_ alloy, which exhibits a yield strength of 297 MPa and an elongation of 8.1% at room temperature. Surperplastic deformation characteristics were also observed in a hot-extruded LPSO Mg-Y-Gd-Zn alloy, which exhibits a maximum elongation of 700% at 470 °C with the strain rate of 1.7 × 10^−4^ s^−1^ [14]. In addition, He et al. [15] reported that the high-cycle fatigue strength of an as-cast LPSO Mg-Gd-Zn-Zr alloy can reach 105 MPa, which is much higher than that (20–80 MPa) of the conventional Mg alloys (AZ91, AZ61 and ZE41).

However, most of the investigations in mechanical behaviors of LPSO-Mg alloys were performed under quasi-static loading conditions. In fact, structural materials are inevitably subjected to dynamic loadings in their practical applications, such as impact or collision. Especially for Mg alloys, they often suffer from a sudden failure in these conditions. As is well known, crack propagation is an important process in materials fracture. To the authors’ knowledge, however, the roles of LPSO phase in dynamic crack propagation behavior of LPSO-Mg alloys are still unclear due to the lack of effective experimental methods. In this work, a novel dynamic four-point bent test was performed on an as-cast Mg_95.5_Y_3_Zn_1.5_ alloy, which contains a large volume fraction of LPSO phase. The aim of this work is to reveal the roles of LPSO phase in crack propagation behavior of the alloy under dynamic loadings.

## 2. Materials and Methods

A Mg_95.5_Y_3_Zn_1.5_ (at.%) ingot was prepared from pure Mg (99.95 wt.%), Zn (99.95 wt.%) and Mg-20Y (wt.%) master alloys in an electric furnace under the protection of a mixed atmosphere of CO_2_ and SF_6_ with the ratio of 99:1. The alloy melt was homogenized at 750 °C for 0.5 h, and then poured into a water-cooled mold with a casting temperature of 720 °C. The chemical compositions of the alloy were determined by inductively coupled plasma atomic emission spectroscopy (ICP-AES, Spectro, Kleve, Germany) and the results were listed in Table 1.

**Figure 1:**
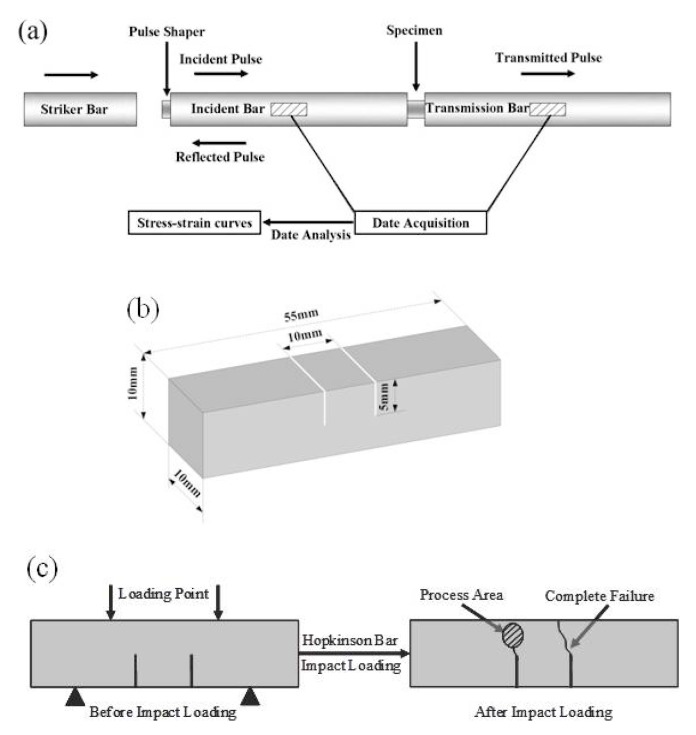
(**a**) Illustration of the Split Hopkinson Pressure Bar; (**b**) Dimensions of the specimens used in dynamic four-point bend tests; (**c**) Mechanism of the dynamic four-point bend tests used in this study.

The microstructure, phase structure and compositions of the as-cast Mg_95.5_Y_3_Zn_1.5_ alloy were characterized by scanning electron microscopy (SEM, JEOL, Tokyo, Japan) equipped with an X-ray energy dispersive spectrometer (EDS) and transmission electron microscopy (TEM, JEOL, Tokyo, Japan). The thin foil samples for the TEM observation were prepared using the argon ion thinning technique. The volume fraction of the LPSO phase was estimated by image analyzer. Dynamic compression tests were performed on a Split Hopkinson Pressure Bar (SHPB, Harbin Engineering University, Harbin, China) at the strain rates of ~1000 s^−1^, ~2000 s^−1^, and ~3000 s^−1^. The set up of the SHPB is shown in Figure 1a. A pulse shaper was placed at the interface between the striker and the incident bar in order to achieve a constant strain rate and a stress state equilibrium for compression experiments at high strain rates. The incident and the reflected strains were measured by the strain gauges glued at the midpoint of the incident bar, while the transmitted strain was measured by the strain gauges glued at the midpoint of the transmission bar. The dynamic stress, strain and strain rate were calculated using one-dimensional stress wave theory. Specimens used for dynamic compression tests were regular cylinders with an 8 mm diameter and 8 mm height. Each test was repeated eight times to ensure the reliability.

To explicitly observe the crack propagation behavior of the alloy under high strain rate, dynamic four-point bend tests were conducted in this study. The specimens for the tests were double-notched, and the dimensions are shown in Figure 1b. The reason for using such specimens can be explained as follows: as shown in Figure 1c, the cracks initiate at both notches during the dynamic compression process. However, only one crack will propagate to complete rupture, while the other crack generates a certain growth, and then it can be arrested immediately by a sudden unloading. Thus, the evolution information with respect to the fracture process, such as crack paths and their interaction with the second phase, is captured in the non-fractured side of the specimen (especially in the process area as shown in Figure 1c).

## 3. Results and Discussion

### 3.1. Microstructure of the As-Cast Mg_95.5_Y_3_Zn_1.5_ Alloy

**Figure 2:**
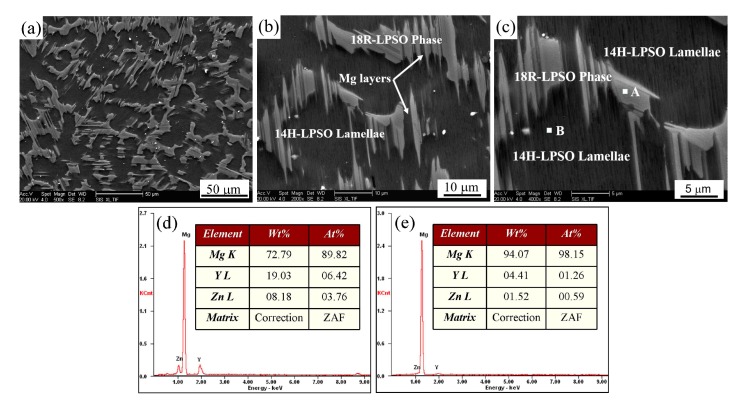
(**a**–**c**) microstructures of the as-cast Mg_95.5_Y_3_Zn_1.5_ alloy; (**d**) energy dispersive spectrometer (EDS) results of the point A in (c); (**e**) EDS results of the point B in (**c**).

Figure 2 shows the microstructure of the as-cast Mg_95.5_Y_3_Zn_1.5_ alloy. As shown in Figure 2a, the alloy mainly consists of α-Mg matrix (with dark contrast) and LPSO phase located at the grain boundaries (with grey contrast). The LPSO phase presents lamellar structures with some Mg layers inside the phase, as indicated by the white arrows in Figure 2b. The volume fraction of the LPSO phase is estimated to be ~38% in the as-cast Mg_95.5_Y_3_Zn_1.5_ alloy. Besides, plenty of fine LPSO lamellae are observed in the interior of the Mg matrix, which arrange uniformly with single direction in a whole grain as shown in Figure 2b,c. Figure 2d,e show the EDS results of the as-cast Mg_95.5_Y_3_Zn_1.5_ alloy. The chemical composition of the phase with grey contrast (marked as A in Figure 2c) and the fine lamellar phase (marked as B in Figure 2c) is Mg-6.42 at.% Y-3.76 at.% Zn and Mg-1.26 at.% Y-0.59 at.% Zn, respectively. The composition of the grey phase is equivalent to that of the LPSO phase (Mg-6 at.% Y-4 at.% Zn) observed in the Mg_97_Y_2_Zn_1_ (at.%) alloys [16]. In the case of the lamellar phase, Y and Zn elements are present in a roughly similar proportion of the LPSO phase. In fact, the LPSO phase located at the grain boundaries exhibits a 18R-type LPSO structure, and the LPSO lamellae in Mg matrix exhibit a 14H-type LPSO structure. The confirmation of the LPSO structures was discussed in the following section.

**Figure 3:**
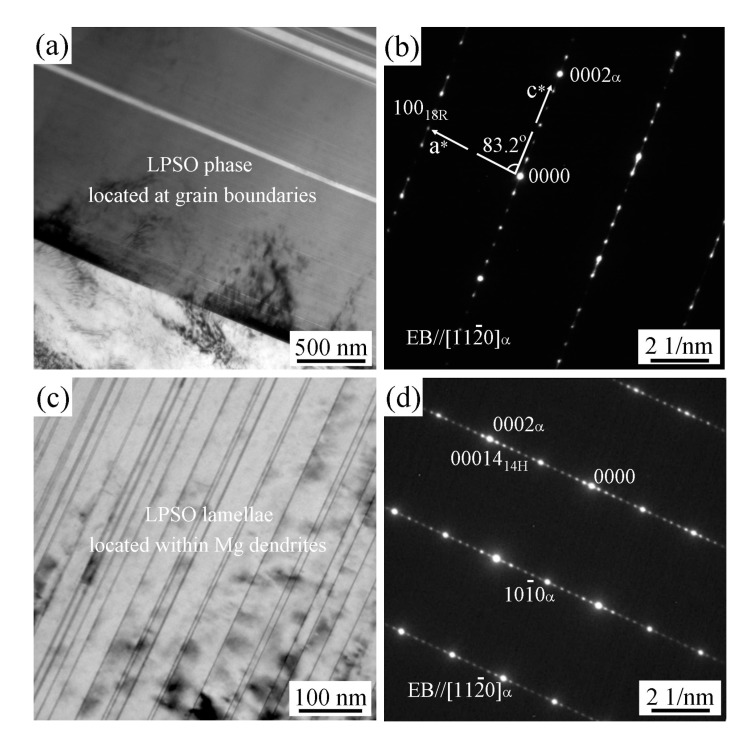
Transmission electron microscopy (TEM) bright field images and selected area electron diffraction (SAED) patterns of the long period stacking ordered (LPSO) phases in the as-cast Mg95.5Y3Zn1.5 alloy: (**a**,**b**) LPSO phase located at the grain boundaries; (**c**,**d**) LPSO lamellae located within the Mg matrix.

Figure 3a,b show the bright-field (BF) TEM image and the corresponding selected area electron diffraction (SAED) pattern of the LPSO phase, which is located at the grain boundaries, respectively. The SAED pattern shows that the phase exhibits a typical 18R LPSO structure with extra diffractions spots appearing at the ±1/6(0002)_α_, ±2/6(0002)_α_…±5/6(0002)_α_ positions [17,18]. In addition, the angle between a* and c* reciprocal vectors is about 83.2° (Figure 3b), which is well consistent with the lattice parameter *β* = 83.25° of 18R-type LPSO structure [19].

The BF TEM image and the corresponding SAED pattern of the fine LPSO lamellae, which is located within the Mg matrix are shown in Figure 3c,d, respectively. It can be clearly seen that the spots of (00014)_14H-LPSO_ and (0002)_α-Mg_ are coincident in the same position. Moreover, small periodic diffraction spots are found at the interval of 1/14 distance between the transmitted spot and the (0002)_α-Mg_ reflection. The results indicated that the fine lamellae phase within the Mg matrix exhibits a 14H-type LPSO structure [20,21].

### 3.2. Mechanical Properties of the As-Cast Mg_95.5_Y_3_Zn_1.5_ Alloy

**Figure 4:**
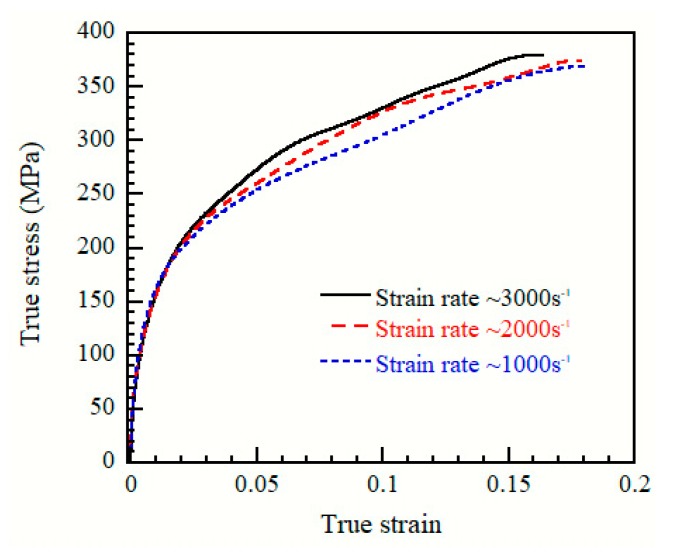
True stress-strain curves of the as-cast Mg_95.5_Y_3_Zn_1.5_ alloy tested at the strain rates of ~1000 s^−1^, ~2000 s^−1^, and ~3000 s^−1^.

The typical true stress-strain curves of the as-cast Mg_95.5_Y_3_Zn_1.5_ alloy tested under dynamic strain rate (1000 s^−1^–3000 s^−1^) loadings are shown in Figure 4. The main mechanical properties are listed in Table 2. The as-cast Mg_95.5_Y_3_Zn_1.5_ alloy exhibits excellent dynamic mechanical properties: for example, the compressive yield stress, the maximum stress and the strain to failure reach up to 190.51 ± 3.52 MPa, 378.32 ± 4.26 MPa and 0.168 ± 0.006, respectively, under the strain rate of ~3000 s^−1^. 

### 3.3. Crack Propagation Behavior of the As-Cast Mg_95.5_Y_3_Zn_1.5_ Alloy under Dynamic Loadings

**Figure 5:**
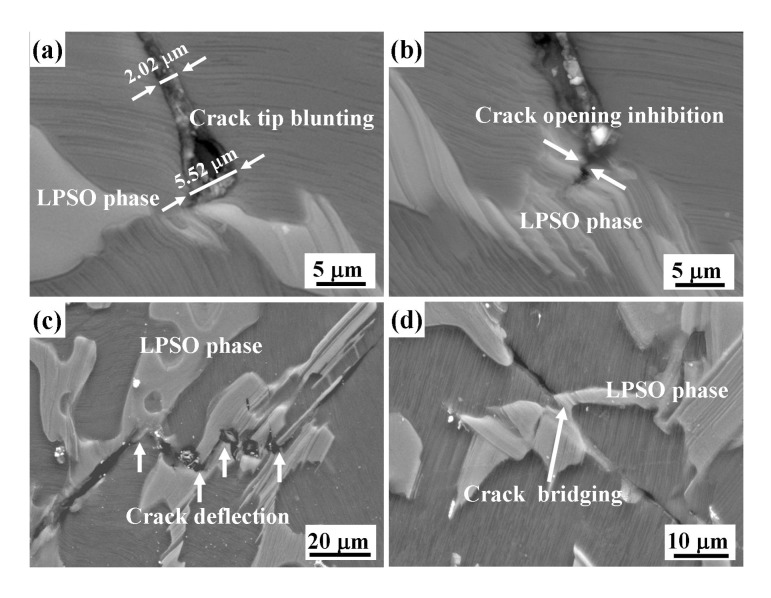
Microstructure of the as-cast Mg_95.5_Y_3_Zn_1.5_ alloy after dynamic deformation at the strain rate of ~3000 s^−1^, showing the relationships between the cracks and LPSO phase: (**a**) Crack tip blunting (the white arrows indicated crack opening displacement); (**b**) Crack opening inhibition; (**c**) Crack deflection; (**d**) Crack bridging.

Dynamic four-point bend tests were conducted to investigate the role of the LPSO phase in crack propagation behavior of the as-cast Mg_95.5_Y_3_Zn_1.5_ alloy. There were four typical interactions between the cracks and the LPSO phase. Firstly, as shown in Figure 5a, the crack opening displacement (COD) (indicated by the white arrows) increased dramatically form 2.02 μm to 5.52 μm as the crack tip grew to the vicinity of the LPSO phase. Meanwhile, the crack tip became obviously round. This strongly indicates that the crack tip was blunted by the LPSO phase. Secondly, as shown in Figure 5b, the crack entered into the LPSO phase. However, the COD sharply decreased as the crack propagated within the LPSO phase, which indicated that the LPSO phase can effectively impede the crack opening. Previous investigations indicated that LPSO phase itself usually possesses many excellent characteristics, such as high micro-hardness [22] and high toughness [23]. Therefore, the LPSO phase can hinder the entry of the cracks to a large extent. Once the cracks go into the LPSO phase, it can be closed just after a short propagation in the LPSO phase.

Thirdly, due to the strongly hindering effect of LPSO phase on the crack propagation, the propagation direction of the crack is sometimes obviously altered, as shown in Figure 5c. At this point, the crack deflection makes the paths of crack propagation complicated, which enlarges the surface area of crack and increases its propagation distance. Therefore, more energy can be consumed by the additional crack surface area and propagation distance. In addition, the stress state (e.g., the stress value, directions) of the crack tip can be changed after the crack deflection. As a result, the driving force of crack propagation is reduced.

Fourthly, as shown in Figure 5d, the crack consistently propagated along its original direction, but it did not fracture the LPSO phase when it encountered the LPSO phase. The LPSO phase just acted as a “bridge” to connect both sides of the crack surfaces. In this situation, the LPSO phase is expected to offer a closure stress, which makes the crack surfaces close to each other. Hence, the crack opening is hindered by the LPSO phase. It is considered that the LPSO phase can toughen the alloy via a “crack bridging” mechanism.

Based on above analysis, the LPSO phase indeed plays a key role in hindering the crack propagation under high strain rate loading, which results in the improvement of the dynamic mechanical properties. On one hand, to overcome the hindering of LPSO phase, the crack propagation needs larger stress. That is to say, the hindering effects of LPSO phase on crack propagation enhance the strength of the alloy. On the other hand, those effects also prevent the alloy from premature fracture and lead to a high ductility.

## 4. Conclusions

In this work, the crack propagation behavior of an as-cast Mg_95.5_Y_3_Zn_1.5_ alloy was investigated under dynamic strain rate loadings. The main results can be summarized as follows:

(1) The as-cast Mg_95.5_Y_3_Zn_1.5_ alloy mainly consists of α-Mg matrix, 18R LPSO phase located at the grain boundaries and 14H LPSO lamellae located within the Mg matrix.
(2) The as-cast Mg_95.5_Y_3_Zn_1.5_ alloy exhibits excellent dynamic mechanical properties: the compressive yield stress, maximum stress and strain to failure are 190.51 ± 3.52 MPa, 378.32 ± 4.26 MPa and 0.168 ± 0.006, respectively, at the strain rate of ~3000 s^−1^.
(3) The LPSO phase plays an important role in hindering dynamic crack propagation, through crack tip blunting, crack opening inhibition, crack deflection and crack bridging.

## Figures and Tables

**Table 1:** Chemical compositions of the studied alloy.

Alloy	Chemical Compositions (at.%)					
	Y	Zn	Fe	Ni	Cu	Mg
Mg_95.5_Y_3_Zn_1.5_	3.4201	1.6328	0.0026	0.0015	0.0010	Bal.

**Table 2:** Mechanical properties of the as-cast Mg_95.5_Y_3_Zn_1.5_ alloy under the strain rate from ~1000 s^−1^ to ~3000 s^−1^.

Alloy	Yield Stress (MPa)	Maximum Stress (MPa)	Strain to Failure (%)	Strain Rate (s^−1^)
Mg_95.5_Y_3_Zn_1.5_	181.62 ± 3.28	365.56 ± 5.16	0.182 ± 0.008	~1000
	186.45 ± 2.90	372.65 ± 4.80	0.176 ± 0.006	~2000
	190.51 ± 3.52	378.32 ± 4.26	0.168 ± 0.006	~3000
